# Open‐shell Poly(3,4‐dioxythiophene) Radical for Highly Efficient Photothermal Conversion

**DOI:** 10.1002/advs.202406800

**Published:** 2024-09-05

**Authors:** Qi Wei, Jiaxing Huang, Qiao Meng, Zesheng Zhang, Sichen Gu, Yuan Li

**Affiliations:** ^1^ State Key Laboratory of Luminescent Materials and Devices Institute of Polymer Optoelectronic Materials and Devices School of Materials Science and Engineering South China University of Technology Guangzhou 510640 P. R. China; ^2^ Faculty of Materials Science MSU‐BIT University Shenzhen 518172 P. R. China

**Keywords:** near‐infrared absorption, open‐shell, organic semiconductor, photothermal conversion, radicals

## Abstract

Open‐shell organic radical semiconductor materials have received increasing attention in recent years due to their distinctive properties compared to the traditional materials with closed‐shell singlet ground state. However, their poor chemical and photothermal stability in ambient conditions remains a significant challenge, primarily owing to their high reactivity with oxygen. Herein, a novel open‐shell poly(3,4‐dioxythiophene) radical PTTO_2_ is designed and readily synthesized for the first time using low‐cost raw material via a straightforward BBr_3_‐demethylation of the copolymer PTTOMe_2_ precursor. The open‐shell character of PTTO_2_ is carefully studied and confirmed via the signal‐silent ^1^H nuclear magnetic resonance spectrum, highly enhanced electron spin resonance signal compared with PTTOMe_2_, as well as the ultra‐wide ultraviolet‐visible‐near nfraredUV–vis–NIR absorption and other technologies. Interestingly, the powder of PTTO_2_ exhibits an extraordinary absorption range spanning from 300 to 2500 nm and can reach 274 °C under the irradiation of 1.2 W cm^−2^, substantially higher than the 108 °C achieved by PTTOMe_2_. The low‐cost PTTO_2_ stands as one of the best photothermal conversion materials among the pure organic photothermal materials and provides a new scaffold for the design of stable non‐doped open‐shell polymers.

## Graphical abstract

The stable poly(3,4‐dioxythiophene) radical PTTO_2_ is reported for the first time, exhibiting a narrow band gap of 0.82 eV and an electron conductivity of 3.11 × 10^−4^ S cm^−1^, surpassing those of closed‐shell P3HT and doped PEDOT. Under irradiation at 1.2 W cm^−2^, the temperature of PTTO_2_ powder can reach 274 °C, representing one of the highest photothermal conversion performances among organic photothermal materials.

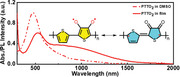

## 1 Introduction

**Figure 1:**
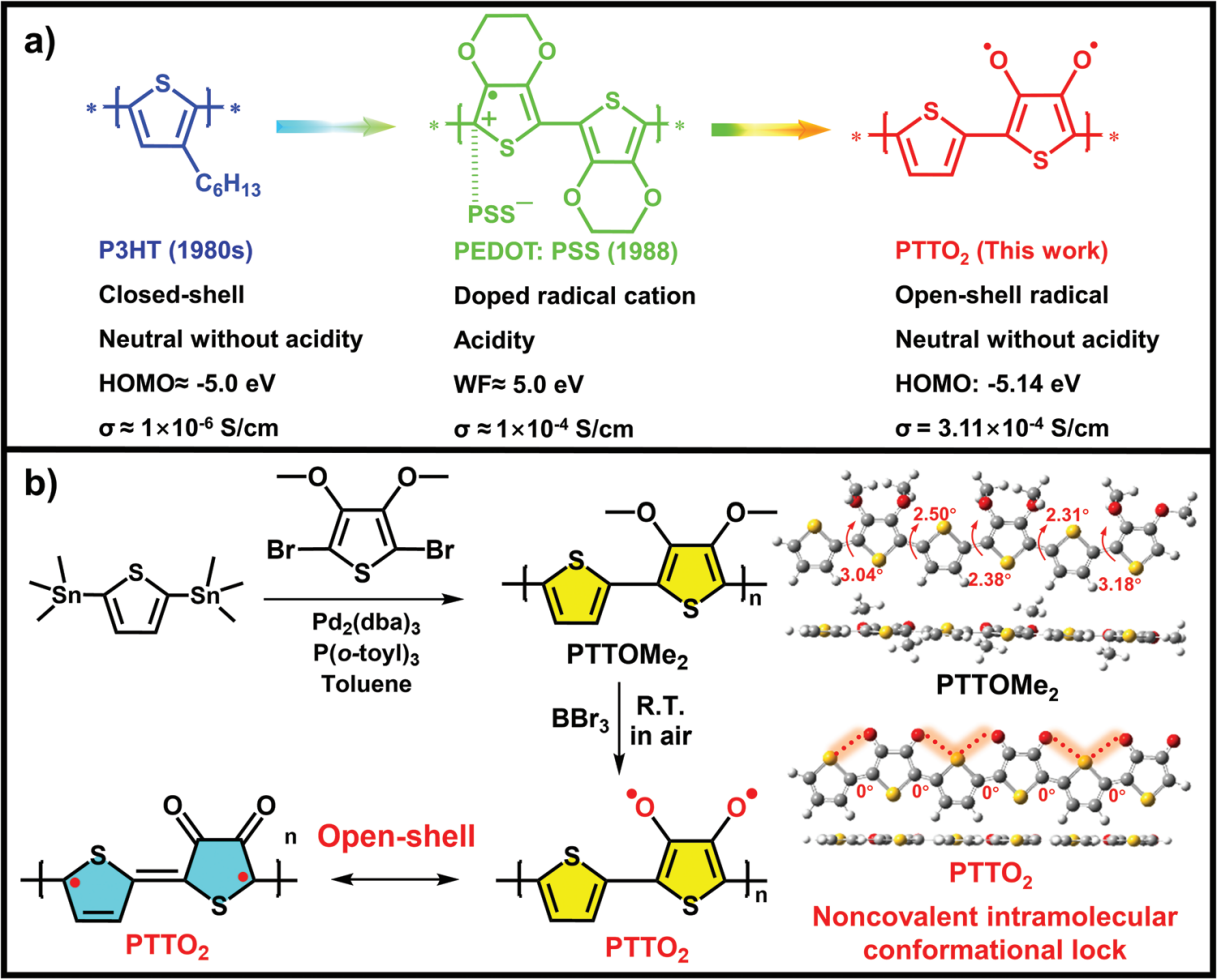
a) The chemical structure and properties of P3HT, PEDOT: PSS and PTTO_2_. b) The synthesis route, the density functional theory calculation on the molecular configuration of PTTOMe_2_ and PTTO_2_, and the non‐covalent intramolecular conformation lock in red dotted line.

Organic semiconductive polymers based on polythiophenes (PTs) have been the subject of extensive research for over three decades owing to their straightforward structure, convenient synthesis, and wide application in various fields.^[^ ^1^, ^2^, ^3^ ^]^ Poly(3‐hexylthiophene) (P3HT) and poly(3,4‐ethylenedioxythiophene): poly(styrenesulfonate) (PEDOT: PSS) stand out as two globally recognized materials among the multitude of organic semiconductive polymers (**Figure** **1**a). P3HT, featuring n‐hexyl side chains on each thiophene ring, shows high solubility, excellent solution‐processibility, relatively high crystallinity and hole mobility, which endow it with great application potential in organic electronic devices, perovskite solar cells, and other fields.^[^ ^4^, ^5^, ^6^, ^7^ ^]^ Throughout the development of PTs, the researchers have devised various strategies to modify P3HT to further enhance its charge transport properties.^[^ ^8^, ^9^ ^]^ However, it is still challenging to achieve high conductivity of P3HT derivatives due to their inherent limitations in hole mobility and charge concentration in these neutral semiconductive polymers. In contrast to P3HT, PEDOT: PSS shows extraordinarily high conductivity as it is a doped polythiophene cationic radical with a low band gap and open‐shell ground state.^[^ ^10^, ^11^, ^12^, ^13^ ^]^ Capitalizing on its exceptional conductivity and solution‐processibility, PEDOT: PSS has shown widespread application as interface material and transparent electrode for organic electronic devices such as organic light‐emitting diodes, organic solar cells, perovskite solar cells, as well as in large‐scale industrial applications in the antistatic, capacitor, printed circuit board and other fields.^[^ ^14^, ^15^, ^16^, ^17^, ^18^ ^]^ However, the water dispersibility, strong acidity caused by the PSS dopant, and relatively low work function (WF) of PEDOT: PSS will inevitably degrade the photoelectric devices to some extent.^[^ ^19^, ^20^ ^]^

It is noteworthy and interesting that the undoped organic radicals materials, such as the non‐conjugated 2,2,6,6‐tetramethylpiperidinooxy (TEMPO)‐based polymer, have displayed high conductivity of 0.28 S cm^−1^ and have been rapidly developed in recent years due to their unique charge transport mechanism.^[^ ^21^, ^22^ ^]^ Moreover, the classical donor‐acceptor (D‐A) materials also exhibited high conductivity.^[^ ^23^, ^24^, ^25^ ^]^ Since 2017, we initially discovered and reported that the classical low‐bandgap D‐A organic semiconductors and P3HT showed widespread open‐shell quinoid‐diradical resonance structure with tunable singlet and triplet ground states, which make them unstable in air.^[^ ^26^, ^27^, ^28^, ^29^, ^30^ ^]^ Nevertheless, the synthesis of these radical materials is relatively complex, their chemical and photothermal stability plays a key role in their practical application due to their high spin concentration and reactivity from strong electronic interactions of radical species.^[^ ^31^, ^32^ ^]^ Herein, we propose a novel design strategy for preparing non‐doped poly(3,4‐dioxythiophene) via introducing the oxygen radical onto the conjugated backbone of PTs (Figure 1a). Intriguingly, the neutral radicals showed high chemical stability without the protection of large steric hindrance groups due to the resonance structure between their oxygen radical and the highly electron‐withdrawing carbonyl structure.^[^ ^33^, ^34^, ^35^, ^36^ ^]^ In addition, stable radical‐based organic materials exhibit unique optical properties, such as near‐infrared (NIR) absorption and non‐radiation decay from a photoelectric excited state, which can sufficiently convert sunlight into heat energy, thus realizing efficient photothermal conversion.^[^ ^37^, ^38^, ^39^ ^]^

In detail, we comprehensively considered the advantages of PTs and organic open‐shell radicals in terms of low cost and convenient synthesis. Accordingly, PTTOMe_2_ was rationally designed and prepared via one‐step stille‐coupling polymerization and PTTO_2_ was readily obtained via demethylation with BBr_3_(99.9%) at room temperature in air (Figure 1b). The polymeric PTTO_2_ radical demonstrated significantly enhanced photothermal conversion performance compared with its precursor PTTOMe_2_. Under the irradiation of an 808 nm laser at a power density of 1.2 W cm^−^^2^, the temperature of PTTO_2_ powder achieved 274 °C within 60 s. Meanwhile, it showed a high‐water evaporation rate of 1.206 kg m^−2^ h^−1^ and solar energy‐to‐vapor efficiency of 83.3% upon one sun irradiation, standing as one of the best pure organic photothermal materials with low cost.

## 2 Results and Discussion

The copolymer PTTOMe_2_ was prepared via the one step simple Stille coupling polymerization using 2,5‐dibromo‐3,4‐dimethoxythiophene and 2,5‐bis(trimethylstannyl)thiophene (Figure 1b) as the raw materials. The PTTO_2_ was obtained via a facile and efficient demethylation reaction of PTTOMe_2_ in a dichloromethane (DCM) solution with BBr_3_ (98%) as a reactant (Figure 1b). The structures of both PTTOMe_2_ and PTTO_2_ were confirmed by ^1^H nuclear magnetic resonance (^1^H‐NMR) spectra (Figures S1 and S2, Supporting Information), Maldi‐tof spectrometry (Figures S3 and S4, Supporting Information) and Fourier transform infrared (FT‐IR) (Figure S5, Supporting Information). It is worth mentioning that PTTO_2_ exhibits a unique property, namely the disappearance of ^1^H NMR spectral signals in solution, which can be attributed to the presence of PTTO_2_ radicals in solution. Figures S3 and S4 (Supporting Information) showed fragment ions with different masses. Notably, the high reactivity of radicals facilitates their facile binding with metal ions. Therefore, when conducting a structural analysis of PTTO_2_, it is essential to consider the potential coordination of PTTO_2_ with metal ions, as this interaction can significantly influence the properties of a compound.^[^ ^40^ ^]^ In addition, we confirmed the successful demethylation of PTTOMe_2_ via FT‐IR as the observation of the disappearance of the methyl signal (3000–2800 cm^−1^). An energy dispersive spectroscopy (EDS) analysis revealed that the atomic percentages of boron (B) and bromine (Br) elements in PTTO_2_ were 0.60% and 0.61%, respectively, suggesting that negligible ion doping occurred during the demethylation process of PTTOMe_2_ to produce PTTO_2_ (Figure S6, Supporting Information). Elemental analysis (EA) was carried out to further verify the experimental results (Table S3, Supporting Information). The measured C, O, and S contents in the PTTOMe_2_ sample are 52.32%, 16.48%, and 28.38%, respectively, closely matching the theoretical values (C, 53.55%; O, 14.27%; S, 28.59%). In the case of PTTO_2_, the measured C, O, and S contents were 48.41%, 19.30%, and 28.09%, respectively, with a notable decrease in the carbon content compared to PTTOMe_2_, aligning with the theoretical prediction of 49.47% for C content.

The thermal stability of PTTOMe_2_ and PTTO_2_ polymers was investigated through thermogravimetric analysis (TGA) to determine the thermal decomposition temperature (*T_d_*). The two powder samples were placed in a crucible under a thermogravimetric analyzer under a nitrogen atmosphere. Thermogravimetric curves were recorded for both materials at a heating rate of 10 °C min^−1^ (as shown in Figure S7, Supporting Information). The 5% weight loss temperature for PTTOMe_2_ and PTTO_2_ were found to be 333 °C and 268 °C, respectively. The relatively low *T_d_* values may be attributed to the relatively low molecular weight of the polymers. Additionally, the differential scanning calorimetry (DSC) results indicated a glass transition temperature (*T_g_*) of 86 °C for PTTOMe_2_. In contrast, PTTO_2_ did not exhibit any clear thermal transitions during the test process, suggesting an absence of both a distinct glass transition and crystallization behavior (Figure S8, Supporting Information).

Density functional theory (DFT) calculations based on Gaussian at a B3LYP/6‐31G (d, p) level were utilized to probe the conformational characteristics of the polymer and the polymer skeleton is represented by two repeat units (Figure 1b). The calculated dihedral angles between adjacent units along the main chain of PTTOMe_2_ are 3.04°, 2.50°, 2.38°, 2.31°and 3.18°, respectively. Besides, the dihedral angle calculation results of PTTO_2_ revealed a strikingly planar structure. According to the reported work, the planar configuration of PTTO_2_ comes from the noncovalent bond effect between sulfur and oxygen of the thiophene backbone. This result can also explain the extremely poor solubility of PTTO_2_.^[^ ^41^, ^42^ ^]^

**Figure 2:**
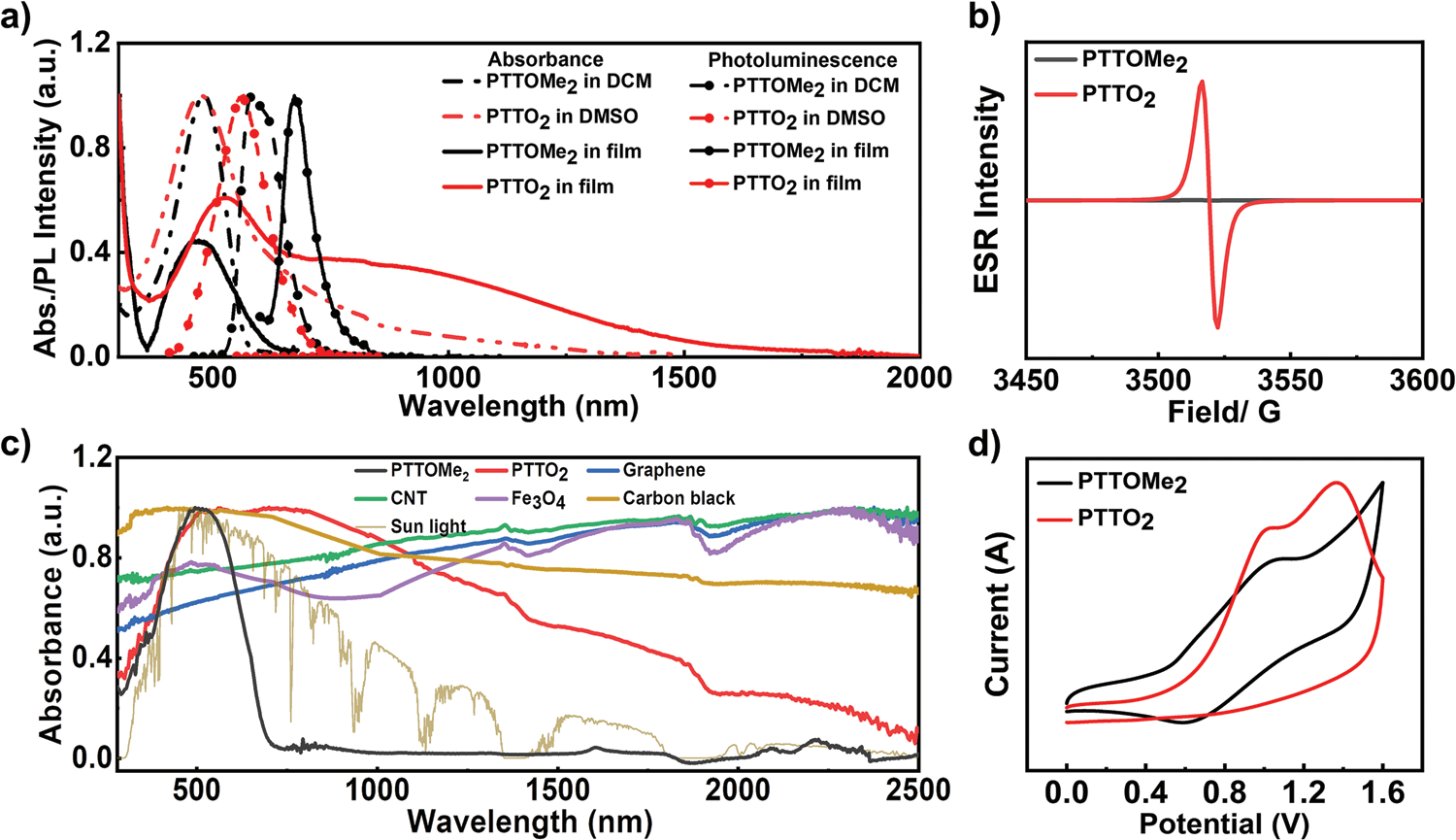
a) UV–vis–NIR absorption spectra and photoluminescence spectra in solution as well as film. b) Electron paramagnetic spectra of the PTTOMe_2_ and PTTO_2_ powder (0.02 mmol). c) UV–vis–NIR spectrum of PTTOMe_2_ and PTTO_2_ in powder compared with inorganic materials (diffuse reflection mode). d) Cyclic voltammetry curves of PTTOMe_2_ (in solution) and PTTO_2_ (in film) with Hg/Hg_2_Cl_2_ electrode as reference. Potential values are reported with the saturated calomel electrode as the reference electrode using the Fc^+^/Fc couple (0.317 V) as an internal standard.

The optical absorption properties of PTTOMe_2_ and PTTO_2_ were studied by UV–vis absorption spectra in **Figure** **2**a. The UV–vis absorption spectra of both PTTOMe_2_ and PTTO_2_ in solution or thin film all displayed typical sharp absorbance bands between 300 and 600 nm, which can be well identified as the absorption of conjugated polythiophene backbone. In addition, the solution of PTTO_2_ in DMSO exhibited a wide absorption band between 600 and 1500 nm, indicating the contribution of the open‐shell PTTO_2_ radical in the solution, which has been similarly reported in previous work.^[^ ^43^, ^44^, ^45^, ^46^ ^]^ Compared with PTTO_2_ in solution state with an absorption edge ≈1380 nm, the film of PTTO_2_ showed an absorption edge approaching 1520 nm, indicating a lower band gap due to the aggregation effect. The characteristic radical peaks observed in the PTTO_2_ film are associated with an aggregation‐induced radical (AIR) effect.^[^ ^26^, ^27^, ^28^, ^29^, ^30^ ^]^ Specifically, the radicals are easily combined with trace water in a solution to form hydroxyl groups, while the quinone radicals are conducive to being produced in a film due to the AIR effect, pancake bond formation, and π–π stacking.^[^ ^29^, ^30^, ^47^ ^]^ Accordingly, the optical bandgaps of PTTOMe_2_ and PTTO_2_ in film are 1.82 and 0.82 eV, respectively. These results indicate that the design of the open‐shell PTTO_2_ is a facile and efficient strategy to decrease the band gap of PT polymers.

The photoluminescence (PL) spectra of PTTOMe_2_ and PTTO_2_ are shown in Figure 2a. In the solution state, the PL spectra of PTTOMe_2_ in DCM and PTTO_2_ in DMSO showed peaks ≈583 and 562 nm, respectively. The PL of PTTO_2_ in the DMSO solution originates from the hydroxyl polymer PTTOH_2_ due to the reaction of PTTO_2_ radicals with the trace water. The spin‐coated film of PTTOMe_2_ showed weak dark‐red emission ≈675 nm, whereas the PL of the corresponding PTTO_2_ radical in the film is quenched. The results are in good agreement with the previous reports indicating that the radical materials usually show extremely low photoluminescence quantum yields (PLQYs) and negligible radiation decay of PTTO_2_ in the solid state.^[^ ^26^, ^27^, ^28^, ^29^, ^30^ ^]^ The nonradiative decay will be largely boosted due to the open‐shell quinoidal radical structure, which will enhance the photothermal conversion efficiency with the promising application potential of PTTO_2_ in the solid state.^[^ ^33^, ^48^ ^]^

To further explore the open‐shell radical structure of PTTO_2_, the radical characters of the two polymers were characterized via the electron spin resonance (ESR) spectrum. Under the same test condition, the powder of the polymer PTTOMe_2_ showed almost none of the paramagnetic signal. In contrast, a significant increase in the ESR signal of PTTO_2_ was detected after the demethylation of the corresponding methoxy precursors (Figure 2b). The hydroxyl groups generated after the demethylation of the red PTTOMe_2_ are readily oxidized into the black PTTO_2_ radicals by oxygen in the air at room temperature, resulting in the production of oxygen radicals and enhanced ESR signal, which is consistent with the results obtained from ^1^H‐NMR, FTIR, EDS, and elemental analysis test.^[^ ^42^, ^43^ ^]^ In general, the high spin concentration of the open‐shell PTTO_2_ is beneficial for the non‐radiative transitions to improve photothermal conversion capability.^[^ ^49^, ^50^ ^]^

Then, we performed solid‐state UV–vis–NIR absorption spectroscopy experiments using the diffuse reflection technique at room temperature, and the results are presented in Figure 2c. PTTO_2_ showed a broader light absorption range compared with that of PTTOMe_2_, owing to the strong electronic tunneling coupling between the neighboring radical molecules.^[^ ^37^ ^]^ Different from the UV–vis absorption spectra in solution, the absorption spectra of the PTTO_2_ extend from 300 to 2500 nm in the powder state (Figure 2c), similar to the graphene, carbon black, and Fe_3_O_4_, covering the entire solar spectrum, indicating that the intermolecular interaction in the solid state can greatly promote its extended wavelength absorption.^[^ ^37^ ^]^ The absorption of PTTO_2_ powder is much broader than those of previous pure organic materials (Figure 4c).^[^ ^35^, ^38^, ^39^, ^44^, ^51^, ^52^, ^53^ ^]^ These results demonstrate that PTTO_2_ powder can absorb most of the UV–Vis and near‐infrared light, which is consistent with its black appearance.

To study the electrochemical characteristics and energy level of the two polymers, the cyclic voltammetry (CV) test was conducted in the air using n‐Bu_4_NPF_6_ as the supporting electrolyte in dry acetonitrile solution and Hg/Hg_2_Cl_2_ as reference electrode (Figure 2d). The highest occupied molecular orbital (HOMO) energy level of each polymer sample is calculated by intercepting the inflection point of the single‐loop CV curve. The HOMO of PTTOMe_2_ and PTTO_2_ are recorded as −4.85 and −5.14 eV, respectively, indicating that the demethylation can efficiently lower the HOMO energy level. Both the solution and powder of PTTO_2_ in the air are stable for several months due to its deeper HOMO level compared with that of PTTOMe_2_. The structure of PTTO_2_ is similar to that of o‐quinone (Figure 1b), and the two resonance carbonyl groups with strong electron acceptor character contribute to its relatively lower HOMO energy level than that of PTTOMe_2_. In the future, we will apply this strategy to introduce electron‐deficient acceptor groups to further reduce the HOMO level and alkyl chains to enhance the solubility and solution‐processability of PTTO_2_‐based low HOMO polymers. Consequently, the conductivity of PTTO_2_ thin film was measured using the four‐probe meter, and the average value of PTTO_2_ was 3.11 × 10^−4^ S cm^−1^ (**Table** **1**), which is two times higher than the 1.26 × 10^−4^ S cm^−1^ of PTTOMe_2_ and comparable with 1 × 10^−4^ S cm^−1^ of PEDOT: PSS‐4083, widely applied in organic electronic devices and other fields. The relatively high electrical conductivity of PTTO_2_ can be understood by the introduction and interaction of oxygen radicals within its planar structure.^[^ ^46^, ^54^, ^55^ ^]^ We propose that the electrical conductivity can be further enhanced by improving the molecule weight by the copolymerization of the (3,4‐dioxythiophene) radical with other highly soluble and conjugated donor and acceptor building blocks in the future.^[^ ^56^, ^57^ ^]^

**Table 1:** Optical and electrochemical properties and energy levels of radicals and corresponding precursors.

Sample	λ_abs_^sol^ (nm)^a)^	λ_abs_^film^ (nm)^b)^	λ_emi_^sol^ (nm)^c)^	λ_emi_^film^ (nm)^d)^	E_g_^opt^ (eV)^e)^	E_ox, onset_ (eV)^f)^	HOMO (eV)^g)^	Conductivity (10^−4^ S cm^−1^)
PTTOMe_2_	569	682	583	675	1.82	0.37	−4.85	1.26
PTTO_2_	1380	1520	562	‐	0.82	0.66	−5.14	3.11

^a)^ λ_abs_ is the wavelength of the absorption edge of samples in solution;

^b)^ λ_abs_ is the wavelength of the absorption edge of samples in thin film;

^c)^ λ_emi_ is the wavelength of the emission peak of samples in solution;

^d)^ λ_emi_ is the wavelength of the emission peak of samples in thin film;

^e)^ E_g_^opt^ = 1240/λabsfilm;

^f)^ From the oxidation onset potential of PTTOMe_2_ solution in DCM and PTTO_2_ film, respectively;

^g)^ E_HOMO_ = (E_ox, onset_ + 4.8 − E_Fc/Fc+_) eV.

**Figure 3:**
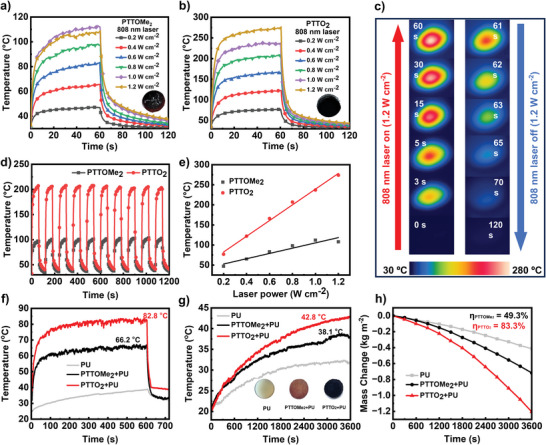
a,b) Photothermal conversion behavior of PTTOMe_2_ and PTTO_2_ powder (20 mg) under 808 nm laser irradiation at different laser powers (0.2–1.2 W cm^−2^) (Insert: Digital photos of PTTOMe_2_ and PTTO_2_ powder, respectively). c) Anti‐photobleaching property of PTTOMe_2_ and PTTO_2_ powder during ten cycles of heating–cooling processes under the 1.2 W cm^−2^ power. d) Linear fitting of platform temperature and power density. e) Infrared thermal images of PTTO_2_ powder under 808 nm laser irradiation (1.2 W cm^−2^). f) Photothermal conversion behavior of PU, PTTOMe_2_+PU and PTTO_2_+PU foams under 1 sunlight irradiation (1.0 kW m^−2^). g) The temperature changes of PU, PTTOMe_2_+PU, and PTTO_2_+PU foams floating on water against sunlight irradiation time. (Insert: Digital photos of PU, PTTOMe_2_+PU, and PTTO_2_+PU foams, respectively). h) Water evaporation curves with PU, PTTOMe_2_+PU and PTTO_2_+PU foams under simulated sunlight with an intensity of 1.0 kW m^−2^.

Next, to evaluate the photothermal conversion performances of PTTOMe_2_ and PTTO_2_, an infrared camera was applied to monitor the temperature change of their powders under 808 nm laser irradiation. **Figure** **3**a,b shows the rising and cooling process of PTTOMe_2_ and PTTO_2_ under the irradiation of 808 nm laser at different power densities. When the power density increased by 0.2 W cm^−2^, the temperature of PTTO_2_ powder rose synchronously by more than 35 °C, which represents a high photothermal conversion performance, showing fast and sensitive photothermal response behavior.^[^ ^44^ ^]^ The temperature of PTTO_2_ powder achieved 274 °C under continuous irradiation at a power density of 1.2 W cm^−2^ power for 1 min (Figure 3c). This result is consistent with the enhanced electronic paramagnetic signal of PTTO_2_ observed in the electron paramagnetic resonance spectrum. It is noteworthy that the relationship between the platform temperature and power density of PTTO_2_ is approximately linear, while PTTOMe_2_ shows irregular changes. The enhanced photothermal conversion can be attributed to the introduction of oxygen radicals in PTTO_2_ through the plasmon resonance effect similar to previous work.^[^ ^58^, ^59^ ^]^ The generation of radicals can promote the process of electroacoustic interconversion, increase the number of phonons, and further convert the optical radiation energy absorbed by electrons into heat energy through thermal radiation, to improve the photothermal conversion efficiency.^[^ ^46^, ^58^, ^59^ ^]^

Then, we also measured the multi‐circle photothermal cycle curves of PTTO_2_ to study the photothermal stability (Figure 3d). During the ten cycles of heating and cooling, the PTTO_2_ showed negligible temperature change, showing good photothermal stability and photobleaching resistance. The outstanding photothermal conversion properties and stability of PTTO_2_ result from its broad NIR absorption, promoted nonradiative decay, and relatively deep HOMO energy level.^[^ ^39^ ^]^ The low‐cost open‐shell radical polymer PTTO_2_ demonstrates great potential for solar thermal conversion compared to other pure organic photothermal conversion materials.^[^ ^51^, ^60^, ^61^, ^62^, ^63^ ^]^

Considering the high efficiency of solar energy collection and broad absorption spectrum from 300 to 2500 nm of PTTO_2_ powder, solar driven water evaporation was carried out to study the photothermal properties of the radical polymers. We employed a commercially available white porous polyurethane (PU) foam with low thermal conductivity as support to establish an efficient interfacial evaporation system by floating the polymer‐loaded PU foam on water.^[^ ^33^ ^]^ The two polymers were loaded (10 mg) inside the PU foam by impregnating pure PU foam in solution and drying, obtaining a brownish‐black PU foam (Figure 3g). The surface temperature of the blank PU foam, PTTOMe_2_+PU, and PTTO_2_+PU were recorded under 1 sun (1 kW m^−2^) irradiation in air and presented in Figure 3f. After irradiating the dry material‐loaded sponge for 10 min, the temperatures of PTTOMe_2_ and PTTO_2_ reached 66.8 °C and 82.8 °C, respectively, exhibiting the fast response to solar light which contributes to the photothermal conversion. In Figure 3g, the temperatures of PTTOMe_2_+PU and PTTO_2_+PU foam floating on the water reached 38.1 °C, and 42.8 °C, respectively, after 1 h of 1 sun irradiation, which is higher than that of the PU foam of 32.3 °C.

**Figure 4:**
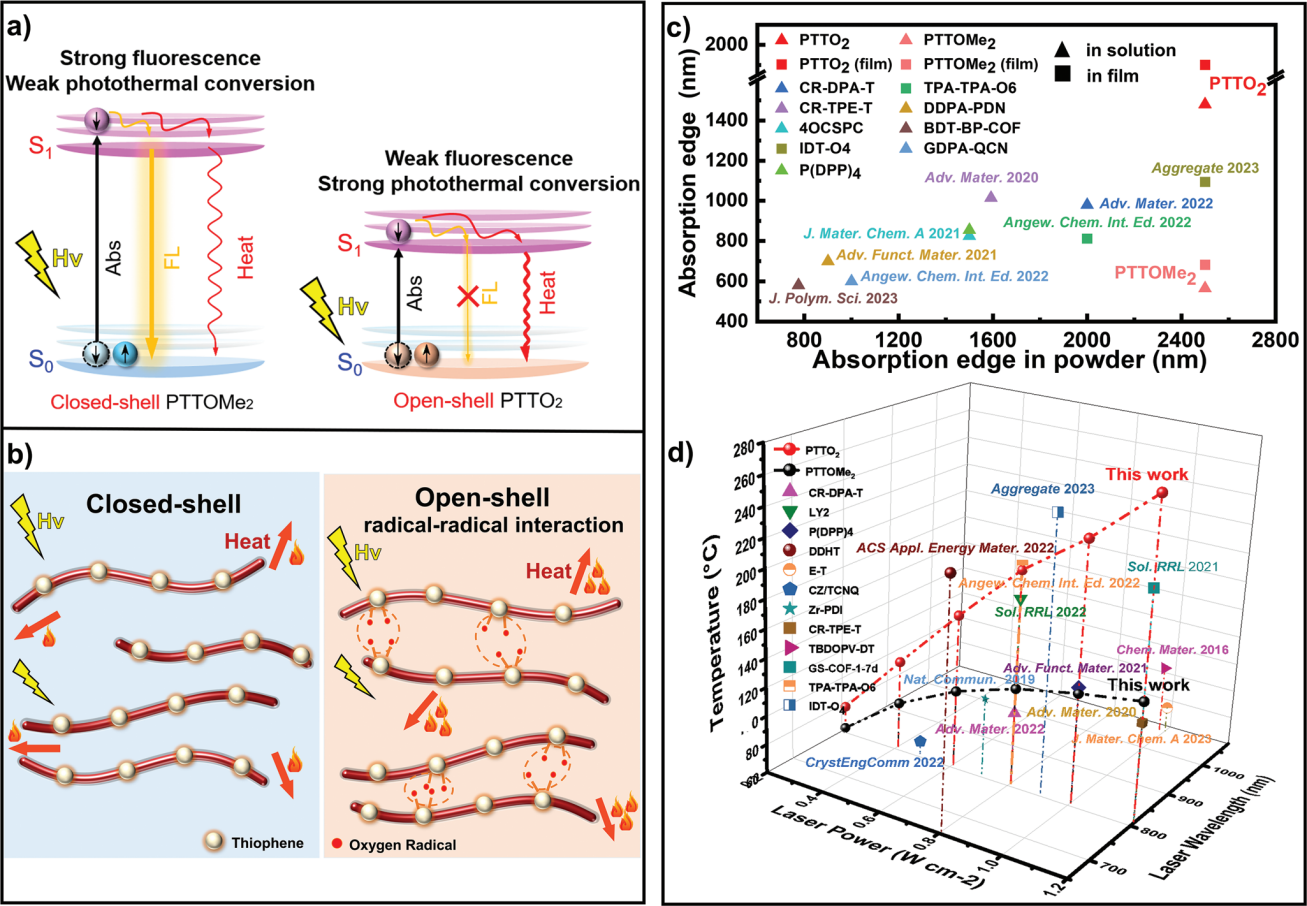
a) The Jablonski diagram shows the difference in energy dissipation of excited states to understand the different photothermal conversion efficiency of PTTOMe_2_ and PTTO_2_. b) Intermolecular chain interaction and photothermal conversion. c) The comparison of absorption edge between pure‐organic small molecules and polymers in powder. d) The temperature change of PTTOMe_2_ and PTTO_2_ under different power densities of the laser. The photothermal conversion performance of PTTOMe_2_ and PTTO_2_ comparing the previously reported pure organic photothermal materials in solid state at different power densities and wavelengths.

The mass change curves of PU foams in water were recorded to assess solar‐driven interfacial water evaporation efficiency (Figure 3h). The PTTO_2_ demonstrates greater mass change and a sharper slope than that of PTTOMe_2_, showing an evaporation rate as high as 1.206 kg m^−2 ^h^−1^ and the solar‐driven water evaporation efficiency (η) of 83.3% under 1 sun illumination (see the detailed calculation process in Supporting Information). These experimental results further confirmed the enhanced photothermal conversion efficiency of the PTTO_2_ radical compared to PTTOMe_2_, because the generation of radicals increases photothermal conversion performance (**Figure** **4**a,b). Compared with the previously reported pure organic photothermal materials, PTTO_2_ exhibits superior photothermal conversion performance (Figure 4c,d; Table S5, Supporting Information). In addition, the synthesis cost of PTTO_2_ is relatively low compared with previously published pure‐organic photothermal conversion materials (Table S4, Supporting Information), indicating the advantages and promising potential of PTTO_2_ for large‐scale production in the future.

## 3 Conclusion

In summary, the two new polymers PTTOMe_2_ and open shell polyradical PTTO_2_ were prepared via two‐step simple reaction using low‐cost raw materials. Compared with PTTOMe_2_, PTTO_2_ exhibited enhanced electronic conductivity of 3.11 × 10^−4^ S cm^−1^, lower HOMO energy level and band gap of 0.82 eV in film, and broadened absorption between 300 and 2500 nm in powder. The continuous irradiation of the PTTO_2_ powder under the power of 1.2 W cm^−2^ in 1 min can elevate its temperature to 274 °C. The enhanced paramagnetic property of PTTO_2_ plays a key factor in its high photothermal conversion performance. This study provides a new poly(3,4‐dioxythiophene) radical backbone for the design and facile preparation of stable polymeric radical materials with potential application prospects in interfacial water evaporation, biomedicine, and other fields.^[^ ^26^, ^64^, ^65^, ^66^ ^]^ More interestingly, this work paved the way for the design and synthesis of low bandgap non‐doped polymers with low‐cost raw materials. Based on this, the synthesis and application of the highly soluble stable open‐shell small molecule and polymers with higher photothermal conversion efficiency based on the 3,4‐dioxythiophene radical are in urgent progress in our lab.^[^ ^26^, ^27^, ^28^, ^67^, ^68^, ^69^, ^70^, ^71^, ^72^, ^73^ ^]^

## Conflict of Interest

The authors declare no conflict of interest.

## Supporting information

Supporting Information

## Data Availability

The data that support the findings of this study are available in the supplementary material of this article.
