# Supplementary material for: Open‐shell Poly(3,4‐dioxythiophene) Radical for Highly Efficient Photothermal Conversion
Source: Adv Sci (Weinh). 2024 Sep 5;11(41):2406800. doi: 10.1002/advs.202406800 (PMC11538641; doi:10.1002/advs.202406800)
Supplement: Supplementary file 1 — Supporting Information [file ADVS-11-2406800-s001.docx]

**Supporting Information**

Open-shell Poly(3,4-dioxythiophene) Radical for Highly Efficient Photothermal Conversion

Qi Wei,^[a]^ Jiaxing Huang,^[a]^ Qiao Meng,^[b]^ Zesheng Zhang,^[a]^ Sichen Gu,*^[b]^ and Yuan Li*^[a]^

[a] Q. Wei, J. Huang, Prof. Y. Li
State Key Laboratory of Luminescent Materials and Devices, Institute of Polymer Optoelectronic Materials and Devices, School of Materials Science and Engineering, South China University of Technology, Guangzhou 510640, P. R. China
E-mail: celiy@scut.edu.cn

[b] Q. Meng, Prof. S. Gu
Faculty of Materials Science, MSU-BIT University, Shenzhen 518172, China
E-mail: sichen.gu@smbu.edu.cn

Keywords: open-shell • photothermal conversion • organic semiconductor • near-infrared absorption • radicals

**Table of Content**

[1. Materials and measurements 3](#_Toc173181507)

[Table S1. Details of materials used in the experiment. 3](#_Toc173181508)

[Table S2. Equipment used in material measurements. 4](#_Toc173181509)

[2. Experimental section 4](#_Toc173181510)

[3. Synthetic route of polymers and characterization 5](#_Toc173181511)

[Scheme S1. Synthetic route of PTTOMe_2_. 5](#_Toc173181512)

[Scheme S2. Synthetic route of PTTO_2_. 6](#_Toc173181513)

[4. ^1^H nuclear magnetic resonance spectra of polymers 7](#_Toc173181514)

[Figure S1 7](#_Toc173181515)

[Figure S2 7](#_Toc173181516)

[5. Maldi-tof spectrum of polymers 8](#_Toc173181517)

[Figure S3 8](#_Toc173181518)

[Figure S4 8](#_Toc173181519)

[6. Fourier transform infrared spectra of polymers 9](#_Toc173181520)

[Figure S5 9](#_Toc173181521)

[7. Energy dispersive spectrometer of PTTO_2_. 10](#_Toc173181522)

[Figure S6 10](#_Toc173181523)

[8. The thermal stability of PTTOMe_2_ and PTTO_2_. 11](#_Toc173181524)

[Figure S7 11](#_Toc173181525)

[Figure S8. 11](#_Toc173181526)

[9. Elemental analysis 12](#_Toc173181527)

[Table S3 12](#_Toc173181528)

[10. The calculation of the efficiency for solar to vapor generation 13](#_Toc173181529)

[11. Comparison of different photothermal conversion materials 14](#_Toc173181530)

[Table S4 14](#_Toc173181531)

[Table S5 18](#_Toc173181533)

[12. Reference 19](#_Toc173181534)

## Materials and measurements.

*Materials:* All the materials were purchased from commercial sources and used as received. Commercially available reagents and chemicals were purchased from Energy Chemical, Guangzhou Chemical Reagent Factory or Alad Chemical Reagents Co. and used without further purification. The details of materials and measurements can be obtained from Table S1 and Table S2. The details of materials and measurements can be obtained from Table S1-S2.

Table S1. Details of materials used in the experiment.

| Materials | CAS | Purity (%) | Producer |
| --- | --- | --- | --- |
| Methylalcohol | 67-56-1 | 99.9 | Guangzhou Chemical Reagent Factory, China |
| Dichloromethane | 67-68-5 | 99.9 |  |
| Dimethylsulfoxide | 75-09-2 | 99.9 |  |
| 2,5-Bis(trimethylstannyl)thiophene | 86134-26-1 | 98 | Energy Chemical,  China |
| BBr_3_ | 10294-33-4 | 99.9 |  |
| N-Bromosuccinimide | 128-08-5 | 98 |  |
| Tris(diben-zylideneacetone)-dipalladium | 51364-51-3 | 98 |  |
| Tri(o-tolyl)phosphine | 6163-58-2 | 98 |  |
| Toluene | 108-88-3 | 99.5 |  |
| 3,4-dimethoxythiophene | 51792-34-8 | 99.9 | Alad Chemical Reagents Co., Ltd, China |

Table S2. Equipment used in material measurements.

| Test method | Equipment specification |
| --- | --- |
| UV-Vis Spectrum (projection mode) | UV-3600 (Shimadzu Co., Japan) |
| Electronic Spin Resonance | Bruker ELEXSYS E500 spectrometer |
| Photothermal Property | 808 nm Fiber Coupled Laser (Model:  MW-GX-808, Changchun Laser Optoelectronics Technology Co., Ltd.); IR thermal camera (FLIR E4) |
| Powder Absorption (diffuse reflection mode) | Lambda 950 spectrophotometer |
| ^1^H-Nuclear Magnetic Resonance | Bruker Avance 500 MHz spectrometer |
| Cyclic Voltammetry | CHI660E, China  [Ag/AgCl (3M KCl solution) electrode;  Carbon-glass electrode; Pt line electrode;Electrochemistry Workstation] |
| FTIR Spectrometer | Bruker Tensor 27 |
| Scanning Electron Microscope  Energy Dispersive Spectrometer | Hitachi Su-70, Hitachi Inc., Japan |
| Element analysis | Elementar UNICUBE |

## Experimental section

**UV-vis measurement.** The UV of the solution was tested at room temperature, and the concentration of the solution was 2×10^-5^ g/mL. The scanning range is set to 300 to 1400nm, with medium speed scanning.

**Electrochemical measurements.** All the CV curves were measured in air in the dry acetonitrile solution containing 0.1 M nBu_4_NPF_6_ as a supporting electrolyte and the scan rate was 0.1 V s^-1^. The carbon-glass electrode, platinum electrode, and saturated calomel electrode were applied as working electrode, counter electrode, and reference electrode, respectively. The test objects in this paper included polymer samples before and after demethylation. In addition, we also tested the electrochemical stability of the polymer samples before and after demethylation for oxidation multiple cycles, and the number of scanning cycles was 20. Potential values are reported with the saturated calomel electrode as the reference electrode using the Fc^+^/Fc couple as an internal standard.

**Electronic spin resonance measurements.** The electronic spin resonance spectra of the totally dried copolymer powder samples (0.02mmol) with the same weight before and after demethylation.

**Photothermal characterizations.** The copolymer powder (20 mg) was paved in a container of 1 cm^2^, and the 808 nm laser irradiation power was adjusted to 0.8 W cm^-2^. Then we adjusted the height of the 808 nm laser irradiator so that it just completely covered the container, and then the infrared radiation thermal camera was aim at the sample container. Then, we turned on the 808 nm laser irradiator. After one minute of exposure, we turn the laser off and keep recording the data. The rest were done in the same manner.

## Synthetic route of polymers and characterization

*Synthesis of the 2,5- dibromo -3,4- dimethoxythiophene.* 2,5- dibromo -3,4- dimethoxythiophene was synthesized according to literature procedure.^[1]^


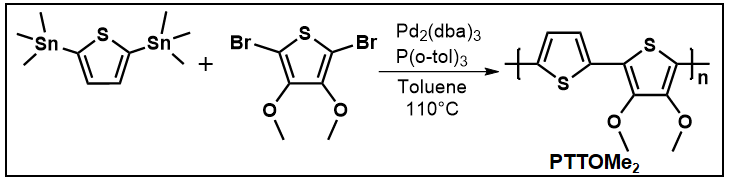


Scheme S1. Synthetic route of PTTOMe_2_.

*Synthesis of the PTTOMe_2_ :* 2,5- bis (trimethyltin) thiophene (500 mg, 1.22 mmol), 2,5- dibromo -3,4- dimethoxythiophene (369 mg, 1.22 mmol), Tris(diben-zylideneacetone)-dipalladium (5 mg, 0.0044 mmol) and Tri(o-tolyl)phosphine (10 mg，0.033mmol) were dissolved in 8 mL toluene. The mixture was heated up to 110 °C and refluxed for 6 h under the nitrogen atmosphere. The crude product was purified by silica gel column chromatography with dichloromethane. Next, the pure product was obtained by recrystallization (CH_3_OH/CHCl_3_) and vacuum drying at 60 °C for 24 hours to afford a red-black solid (150 mg, 54.5 %).


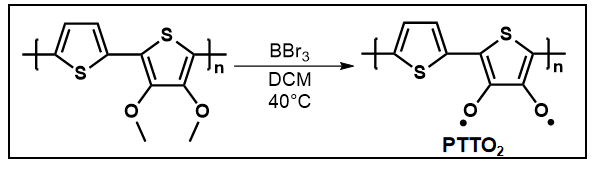


Scheme S2. Synthetic route of PTTO_2_.

*Synthesis of the PTTO_2_:* PTTOMe_2_ (50 mg) was dissolved in 2 mL dewatering dichloromethane. The precursor was cooled to -78 °C by ethanol in the cold trap. Then, 2 mL 1M BBr_3_ (2 mmol) solution was added dropwise. The mixture was then stirred and heated up to 40 °C for 12 h with precipitation of the solid product. Next, methanol was added dropwise into the reacting solution to quench BBr_3_.After dropping the solution into deionized water, the solids can be separated by filtration and the crude product was purified by washing with H_2_O and CH_2_Cl_2_ successively. The complete demethylation product was obtained as thin-layer chromatography (TLC) had no impurities with an eluent of dichloromethane. The product was sent to vacuum drying at 60 °C for 24 hours to give a black PTTO_2_ powder (42mg, 96%).

## 4. ^1^H nuclear magnetic resonance spectra of polymers


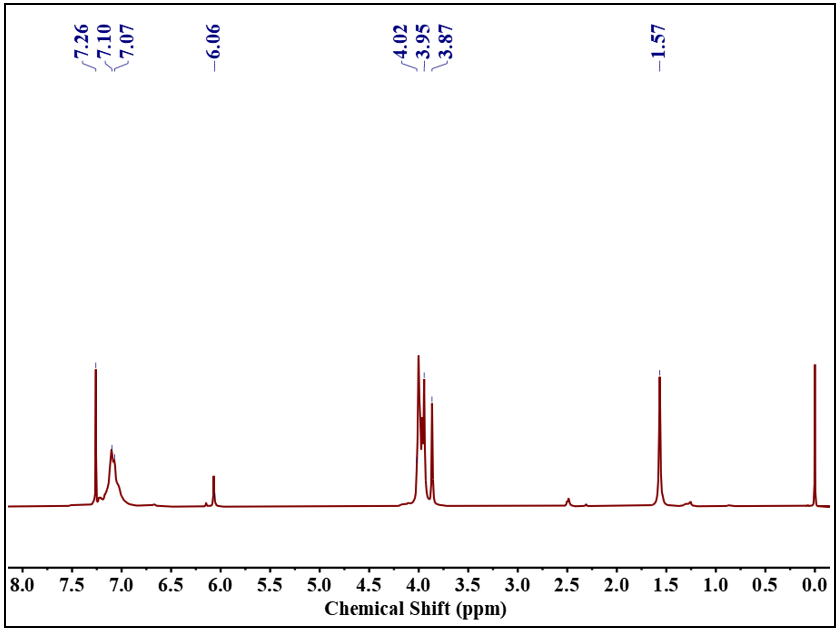


Figure S1 ^1^H NMR spectrum of PTTOMe_2_ in CDCl_3_.


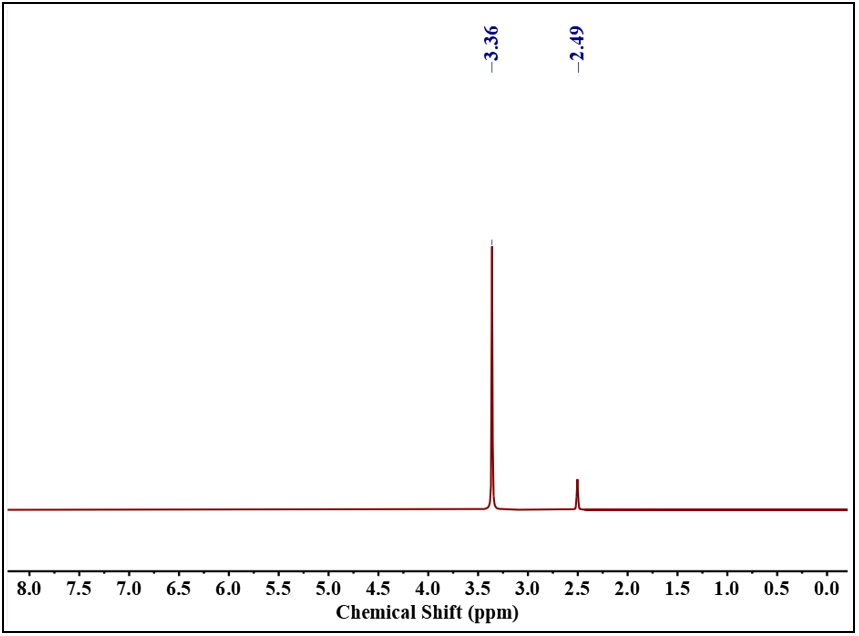


Figure S2 ^1^H NMR spectrum of PTTO_2_ in DMSO-d_6_.

## 5. MALDI-TOF spectrum of polymers


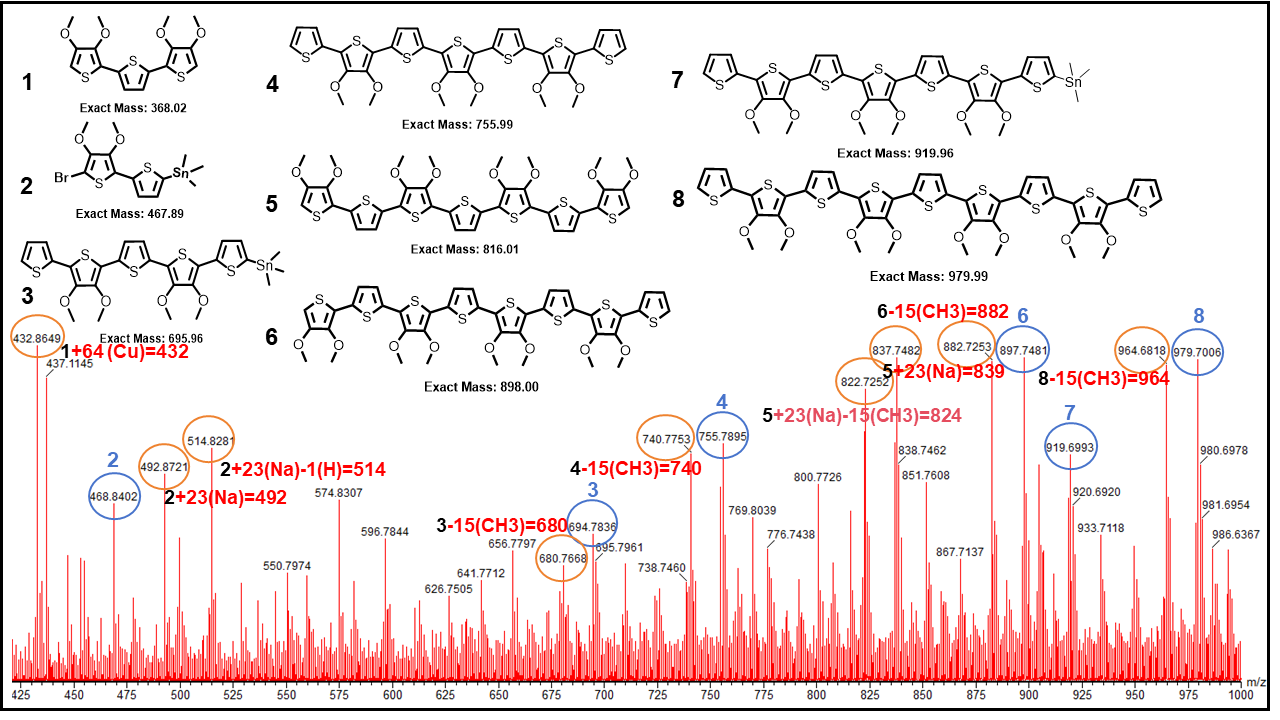


Figure S3 MALDI-TOF spectrum of the PTTOMe_2_.


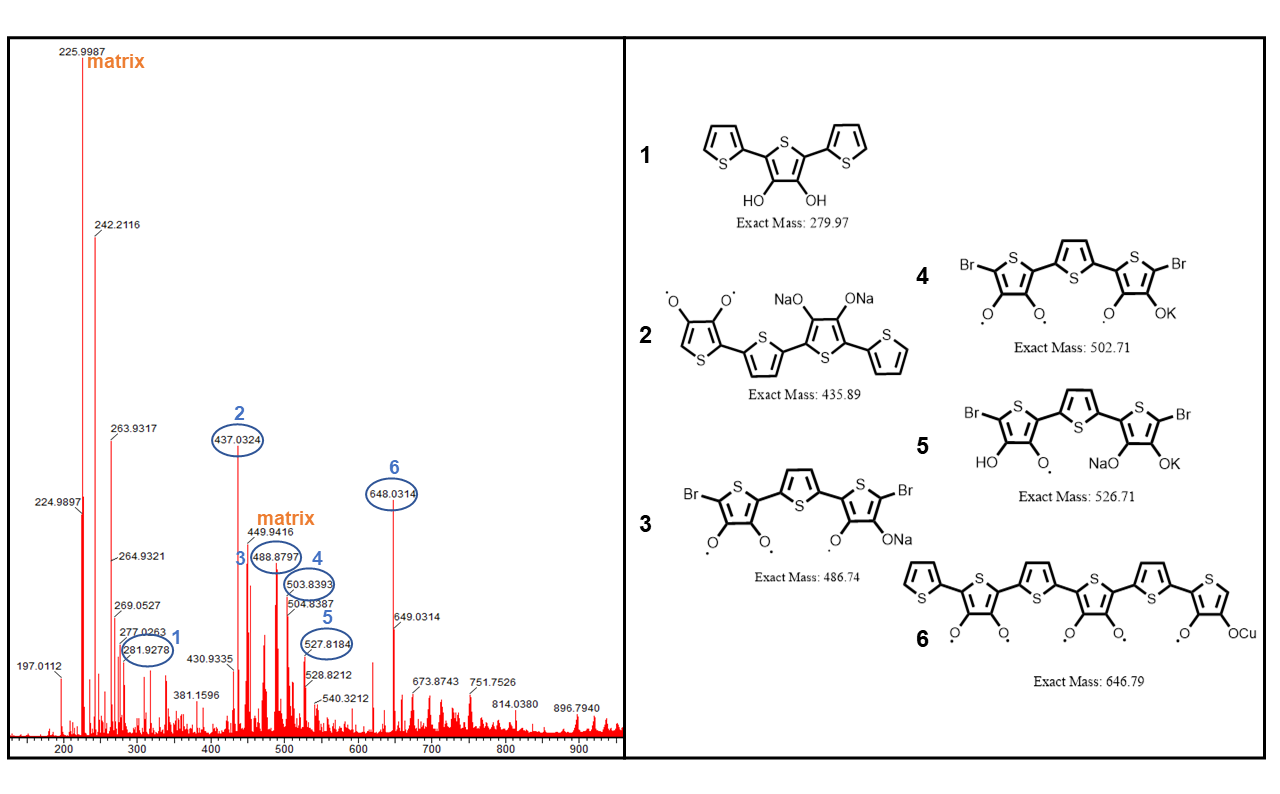


Figure S4 MALDI-TOF spectrum of the PTTO_2_.

## 6. Fourier transform infrared spectra of polymers


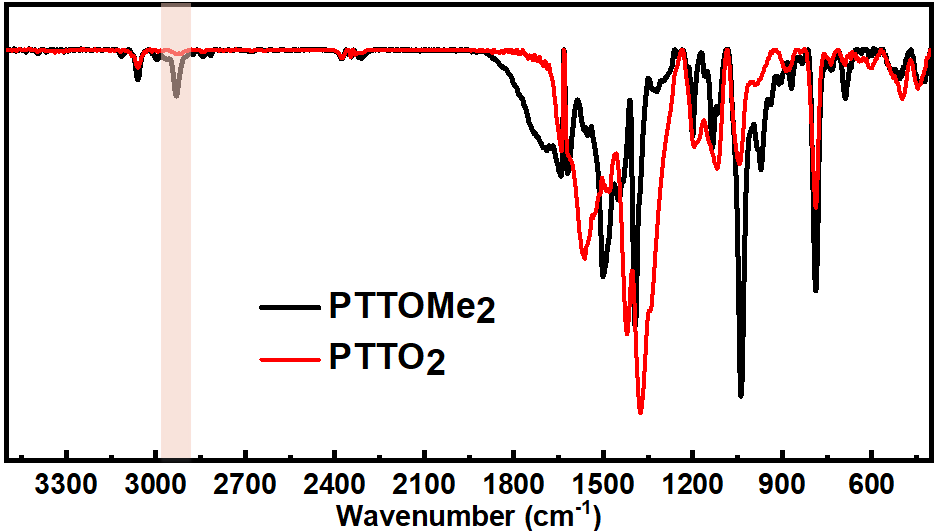


Figure S5 FT-IR spectra of PTTOMe_2_ and PTTO_2_.

## 7. Energy dispersive spectrometer of PTTO_2_.


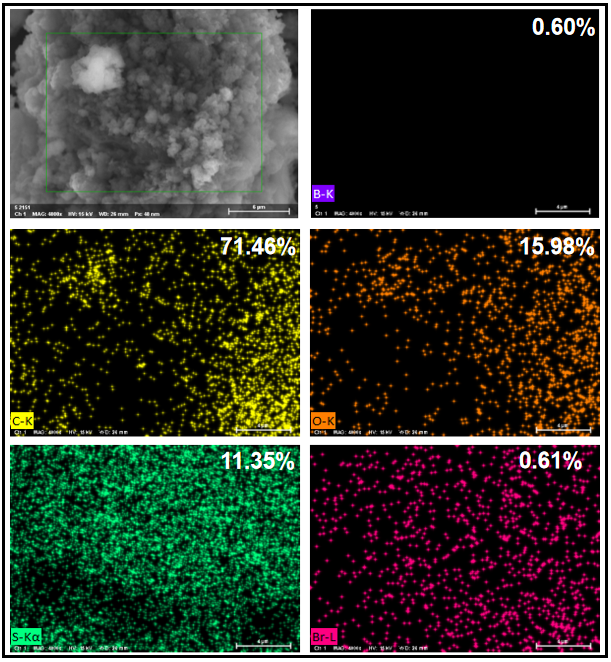


Figure S6 The elemental mapping of B, C, O, S and Br of PTTO_2_.

## 8. The thermal stability of PTTOMe_2_ and PTTO_2_.


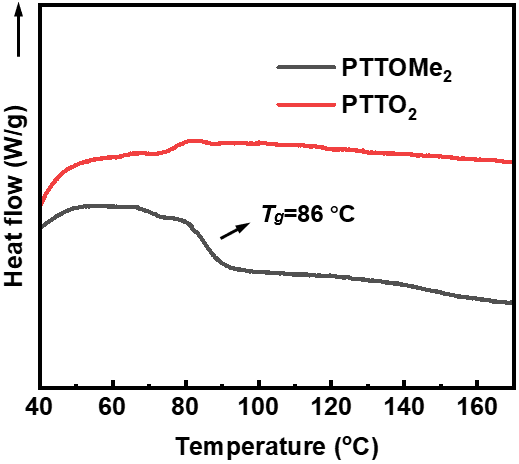


Figure S7. TGA curves of PTTOMe_2_ and PTTO_2_ in N_2_ at a heating rate of 20 °C min^-1^**.**


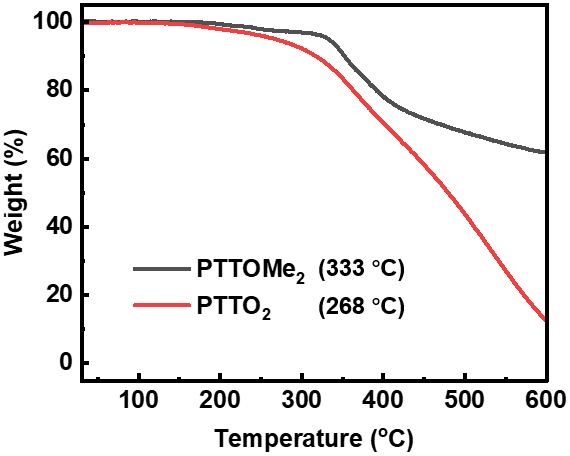


Figure S8. DSC curves of PTTOMe_2_ and PTTO_2_ in N_2_ at a heating rate of 10 °C min^-1^ .

## 9. Elemental analysis

Table S3 Contents of elements C, H, N, S and O in the compounds.

| Element | C (%) | H (%) | N (%) | S (%) | O (%) |
| --- | --- | --- | --- | --- | --- |
| PTTOMe_2_ | 52.32 | 3.22 | 0 | 28.38 | 16.48 |
| PTTO_2_ | 48.41 | 2.55 | 0.0615 | 28.09 | 19.30 |

## 10. The calculation of the efficiency for solar to vapor generation

The conversion efficiency η of solar energy in photothermal assisted water evaporation was calculated as the following formula：

$$\eta= {\dot{m}h_{LV}}/{∁opt}P_{0}$$

Where ṁ refers to the mass flux (evaporation rate) of water, *h_LV_* refers to the total liquid-vapor phase-change enthalpy, Q is the energy provided to heat the system from the initial temperature T_0_ to a final temperature T, $\Delta h_{\mathrm{vap}}$ is the latent heat of vaporization of water, P_0_ is the nominal solar irradiation value of 1 kW m^-2^, and C_opt_ represents the optical concentration (C_opt_ = 1).

$$Q=C_{liquid}\times\left( T-T_{0} \right)$$

$$\Delta h_{vap}=Q_{1}+\Delta h_{vap}+Q_{2}$$

$$h_{LV}=Q+\Delta h_{vap}$$

$$Q_{1}=C_{liquid}\times\left( 100-T \right)$$

$$Q_{2}=C_{vapor}\times\left( T-100 \right)$$

In this paper, C_liquid_, the specific heat capacity of liquid water is a constant of 4.18 J/g ^o^C. C_vapor_, the specific heat capacity of water vaper is a constant of 1.865 J/g ^o^C. $\Delta h_{100}$ is the latent heat of vaporization of water at 100 ^o^C, taken to be 2260 kJ/kg.

**PTTO_2_:**

$$Q=C_{liquid}\times\left( T-T_{0} \right)=4.18\times\left( 42.8-20.5 \right)=93.214 kJ/kg$$

$$\Delta h_{vap}=Q_{1}+\Delta h_{100}+Q_{2}=4.18\times\left( 100-42.8 \right)+2260+1.865\times\left( 42.8-100 \right)=2392.418 kJ/kg$$

$$h_{LV}=Q+\Delta h_{vap}=93.214+2392.418=2485.628 kJ/kg$$

$$\eta= {\dot{m}h_{LV}}/{∁opt}P_{0}={1.206\times2485.628}/{1\times}1=83.3\%$$

## 11. Comparison of different photothermal conversion materials

Table S4 The cost of various photothermal conversion materials.

| Materials | Molecular structure | Cost^[a]^ (¥/g) | Ref. |
| --- | --- | --- | --- |
| PTTOMe_2_ | 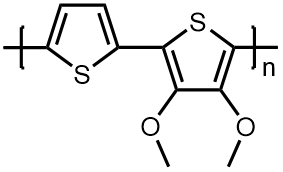 | **About 25** | This work |
| PTTO_2_ | 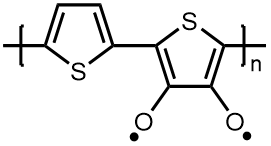 | **About 30** | This work |
| P3HT |  | **> 1000** | ^[2]^ |
| CR-DPA-T |  | **> 350** | ^[3]^ |
| CR-TPE-T |  | **> 350** | ^[4]^ |
| LY2 |  | **> 1100** | ^[5]^ |
| P(DPP)4 |  | **> 500** | ^[6]^ |
| 4OCSPC |  | **> 990** | ^[7]^ |
| PI/CNT |  | **> 120** | ^[8]^ |
| GDPA-QCN |  | **>150** | ^[9]^ |
| TPyP |  | **>210** | ^[10]^ |
| E-T |  | **> 389** | ^[11]^ |
| DDPA-PDN |  | > **150** | ^[12]^ |
| DDHT |  | > **35** | ^[13]^ |
| TBDOPV-DT |  | > **500** | ^[14]^ |
| TPA-TPA-O_6_ |  | > **300** | ^[15]^ |
| 2TP-BBT |  | > **2000** | ^[16]^ |
| BQE |  | > **800** | ^[17]^ |
| ATT |  | > **1000** | ^[18]^ |

[a] Statistics here are the average market price of a reactant during the reaction.

Table S5 The maximum temperature comparison of various photothermal functional materials under different laser power and laser wavelength.

| Materials | Laser power  (W cm^-2^) | Laser wavelength (nm) | | Maximum Temperature (°C) | Reference |
| --- | --- | --- | --- | --- | --- |
| PTTO_2_ | 1.2 | 808 | | 274 | **This**  **work** |
|  | 1.0 |  |  | 237 |  |
|  | 0.8 |  |  | 207 |  |
| PTTOMe_2_ | 1.2 | 808 | | 108 |  |
|  | 1.0 |  |  | 112 |  |
|  | 0.8 |  |  | 98 |  |
| CR-DPA-T | 0.8 | 808 | | 110 | ^[3]^ |
| CR-TPE-T | 1.2 | 808 | | 129 | ^[4]^ |
| LY2 | 0.8 | 808 | | 188 | ^[5]^ |
| P(DPP)_4_ | 1.0 | 808 | | 140 | ^[6]^ |
| CZ/TCNQ | 0.48 | 808 | | 69 | ^[19]^ |
| TPyP | 1.0 | | 730 | 156 | ^[10]^ |
| E-T | 1.0 | 1064 | | 78 | ^[11]^ |
| DDPA-PDN | 0.9 | 655 | | 201 | ^[12]^ |
| DDHT | 0.8 | 655 | | 227 | ^[13]^ |
| TBDOPV-DT | 0.98 | 1064 | | 107 | ^[14]^ |
| PT-N-COF | 1.8 | 808 | | 94 | ^[20]^ |
| Zr-PDI | 0.7 | 808 | | 114 | ^[21]^ |
| GS-COF-1-7d | 1.2 | 808 | | 216 | ^[22]^ |

## 12. Reference

[1] F. Zhang, Z. Wang, H. Zhu, S. Wang, X. Li, *Org. Electron.* **2019**, *71*, 194.

[2] L. Wang, G. Xi, Z. Chen, Q. Wang, J. Liu, R. Zhang, T. Jia, X. Zhao, *J. Solid State Chem.* **2023**, *324*, 124081.

[3] J. Sun, E. Zhao, J. Liang, H. Li, S. Zhao, G. Wang, X. Gu, B. Z. Tang, *Adv. Mater.* **2022**, *34*, e2108048.

[4] G. Chen, J. Sun, Q. Peng, Q. Sun, G. Wang, Y. Cai, X. Gu, Z. Shuai, B. Z. Tang, *Adv. Mater.* **2020**, *32*, e1908537.

[5] W. Liang, J. Huang, W. Zhu, Y. Li, *Sol. RRL* **2022**, *6*, 2200400.

[6] X. Zhang, Y. Li, Z. Chen, P. Li, R. Chen, X. Peng, *Dyes Pigm.* **2021**, *192*, 109460.

[7] X. Han, Z. Wang, M. Shen, J. Liu, Y. Lei, Z. Li, T. Jia, Y. Wang, *J. Mater. Chem. A* **2021**, *9*, 24452.

[8] K. Lan, Y. Deng, A. Huang, S.-Q. Li, G. Liu, H.-L. Xie, *Polymer* **2022**, *256*, 125177.

[9] J. Liu, Y. Cui, Y. Pan, Z. Chen, T. Jia, C. Li, Y. Wang, *Angew Chem. Int. Ed. Engl.* **2022**, *61*, e202117087.

[10] Y. Zhang, H. Yan, X. Wang, Z. Zhang, F. Liu, S. Tu, X. Chen, *RSC Adv.* **2022**, *12*, 28997.

[11] H. Li, H. Li, L. Zou, Q. Li, P. Chen, X. Quan, K. Deng, C. Sheng, J. Ji, Q. Fan, Z. Xu, J. Wan, *J. Mater. Chem. A* **2023**, *11*, 2933.

[12] Y. Cui, J. Liu, Z. Li, M. Ji, M. Zhao, M. Shen, X. Han, T. Jia, C. Li, Y. Wang, *Adv. Func. Mater.* **2021**, *31*, 2106247.

[13] M. Zhao, Y. Zhu, Y. Pan, Y. Wang, T. Xu, X. Zhao, T. Jia, Z. Zhang, Z. Chen, *ACS Appl. Energy Mater.* **2022**, *5*, 15758.

[14] Y. Cao, J. Dou, N. Zhao, S. Zhang, Y. Zheng, J. Zhang, J. Wang, J. Pei, Y. Wang, *Chem. Mater.* **2016**, *29*, 718.

[15] Z. Su, Y. Jin, H. Wang, Z. Li, L. Huang, H. Wang, *ACS Appl. Energy Mater.* **2022**, *5*, 11915.

[16] T. Jia, R. Zhang, L. Wang, J. Liu, M. Nan, S. Qi, S. Liu, N. Jin, Y. Pan, *J. Mater. Chem. A* **2023**, *11*, 26164.

[17] J. Li, L. Wang, C. Zhang, H. Wang, Y. Pan, S. Li, X.-K. Chen, T. Jia, K. Wang, *Angew Chem. Int. Ed. Engl.* **2024**, *63*, e202402726.

[18] P. Han, H. Xu, G. Zhang, A. Qin, B. Z. Tang, *Angew Chem. Int. Ed. Engl.* **2024**, https://doi.org/10.1002/anie.202406381

[19] P. Shi, X. Liu, X. Dai, T. Lu, J. Chen, *CrystEngComm* **2022**, *24*, 4622.

[20] Y. Zhang, G. Wu, H. Liu, R. Tian, Y. Li, D. Wang, R. Chen, J. Zhao, S. Liu, Z. Li, Y. Zhao, *Mater. Chem. Front.* **2021**, *5*, 6575.

[21] B. Lu, Y. Chen, P. Li, B. Wang, K. Mullen, M. Yin, *Nat. Commun.* **2019**, *10*, 767.

[22] Z. Chen, Y. Su, X. Tang, X. Zhang, C. Duan, F. Huang, Y. Li, *Sol. RRL* **2021**, *5*, 2100762.
